# Sweetpotato cultivars responses to interactive effects of warming, drought, and elevated carbon dioxide

**DOI:** 10.3389/fgene.2022.1080125

**Published:** 2023-01-04

**Authors:** Shasthree Taduri, Raju Bheemanahalli, Chathurika Wijewardana, Ajaz A. Lone, Stephen L. Meyers, Mark Shankle, Wei Gao, K. Raja Reddy

**Affiliations:** ^1^ Department of Plant and Soil Sciences, Mississippi State University, Mississippi State, MS, United States; ^2^ Department of Biotechnology, Kakatiya University, Warangal, India; ^3^ Department of Horticulture and Landscape Architecture, Purdue University, West Lafayette, IN, United States; ^4^ Pontotoc Ridge-Flatwoods Branch Experiment Station, North Mississippi Research and Extension Center, Mississippi State University, Pontotoc, MS, United States; ^5^ USDA UVB Monitoring and Research Program, Natural Resource Ecology Laboratory, Department of Ecosystem Science and Sustainability, Colorado State University, Fort Collins, CO, United States

**Keywords:** CO_2_, climate change, drought stress, high temperature, interactive stresses, storage roots

## Abstract

Plants are sensitive to changes projected in climates, such as elevated carbon dioxide (eCO_2_), high temperature (T), and drought stress (DS), which affect crop growth, development, and yield. These stresses, either alone or in combination, affect all aspects of sweetpotato plant growth and development, including storage root development and yield. We tested three sweetpotato cultivars (Beauregard, Hatteras, and LA1188) responses to eight treatments (Control, DS, T, eCO_2_, DS + T, T + eCO_2,_ DS + eCO_2,_ DS + T + eCO_2_). All treatments were imposed 36 days after transplanting (DAP) and continued for 47 days. Treatments substantially affected gas exchange, photosynthetic pigments, growth, and storage root components. Cultivars differed considerably for many of the measured parameters. The most significant negative impact of DS was recorded for the shoot and root weights. The combination of DS + T had a significant negative effect on storage root parameters. eCO_2_ alleviated some of the damaging effects of DS and high T in sweetpotato. For instance, eCO_2_ alone or combined with DS increased the storage root weights by 22% or 42% across all three cultivars, respectively. Based on the stress response index, cultivar “Hatteras” was most tolerant to individual and interactive stresses, and “LA 1188” was sensitive. Our findings suggest that eCO_2_ negates the negative impact of T or DS on the growth and yield of sweetpotato. We identified a set of individual and interactive stress-tolerant traits that can help select stress cultivars or breed new lines for future environments.

## 1 Introduction

Sweetpotato [*Ipomoea batatas* (L.) Lam.], a member of the Convolvulaceae, is the seventh most important food crop globally with more than 100 million tons annual production, grown over 8.62 million ha and in over 100 countries (FAO, 2016). In the US, the harvested sweetpotato area had increased from 36,219 ha in 1990 to 63,455 ha in 2020 (https://quickstats.nass.usda.gov). Simultaneously, the average sweetpotato yield increased from 19.5 to 27.1 tons ha^−1^. The sweetpotato production contributed c.a. $500 million to the United States economy in 2012 (USDA, 2016). Sweetpotato commercial output is concentrated in the Southeast region and California within the United States. Although sweetpotato can adapt to various environmental conditions, its productivity can be reduced by specific abiotic stresses. Environmental conditions such as drought stress (DS), high temperature (T), and elevated carbon dioxide (eCO_2_), either alone or in combination, can affect both the plant health and yields of sweetpotato (Gajanayake et al., 2014; Liu, 2017; Wijewardana et al., 2018; Heider et al., 2021).

Plants are sensitive to environmental changes such as atmospheric CO_2_, T, and DS, which affect crop growth, development, and yield. The average global temperature on Earth has increased by about 1.1°C since 1880 [https://www.climate.gov/news-features/understanding-climate/climate-change-atmospheric-carbon-dioxide]. Moreover, it is predicted by climatologists that by the end of the 21st century, the temperature is likely to increase by another 3°C–4°C, and the atmospheric CO_2_ will increase from 410 ppm to 900 ppm (Birch, 2014; Intergovernmental Panel on Climate Change, 2018). Under such scenarios, it is necessary to quantify the impacts of climate change on current crop production to meet the demands of the burgeoning world population. High temperature is one of the most critical stress factors affecting plant growth, development, and yield (Gajanayake et al., 2014; Bheemanahalli et al., 2016, 2019; Reddy et al., 2017). Recently, studies showed the adverse effects of temperature on sweetpotato early establishment and storage root initiation. High T significantly reduced storage root production by 80%–90% in sweetpotato and formed more pencil roots (Gajanayake et al., 2014; Wijewardana et al., 2018). High T can cause a reduction in the formation and growth of storage roots through the changes in phytohormone synthesis and activation and the alteration of gas exchange and other physiological processes (Kooman and Haverkort, 1995). However, most of these studies have focused on one or two stress factors. They have given little attention to storage root formation and morpho-physiological changes during crop growth and development.

Along with high T, DS is another essential environmental stress factor affecting crop production globally (Saini and Westgate, 1999; Birch, 2014). Optimum leaf water potential is vital for normal physiological activities and membrane transport processes in plants, and thus, it is essential for plant growth and development. A lack of sufficient soil moisture limits photosynthetic activity, ultimately affecting storage root development, yield, and quality (Gollifer, 1980; van Heerden and Laurie, 2008; Lewthwaite and Triggs, 2012; Laurie et al., 2014). The storage root bulking of sweetpotato was affected by combination of stresses (Lewthwaite and Triggs, 2012; Omotobora et al., 2014; Heider et al., 2021). DS decreased photosynthesis during the initial vegetative growth phase by lowering stomatal and mesophyll conductance or causing oxidative damage to the chloroplast (Flexas et al., 2004; Medeiros et al., 2016). Severe drought impairs the regeneration of ribulose bisphosphate (RuBP) and decreases the activity of ribulose 1, 5-bisphosphate carboxylase/oxygenase (Rubisco), resulting in lower photosynthesis (Bota et al., 2004). Early studies evaluated photosynthesis variability under water stress using sweetpotato varieties consumed in Africa (van Heerden and Laurie, 2008), but the association between storage root initiation and yield was not determined.

Increases in atmospheric CO_2_ concentration will, directly and indirectly, affect crop plants at different developmental stages. eCO_2_ generally increases plant productivity, photosynthesis, and water-use efficiency (Drake et al., 1997; Fleisher et al., 2008; Baker et al., 1989; Kimball, 1983). The yield of crops will increase under eCO_2_, with productivity rising from 15% to 41% for C_3_ plants and 5%–10% for C_4_ crops (Kimball, 1983; Lotze-Campen and Schellnhuber, 2009; Brand et al., 2016). Even though eCO_2_ positively impacts plant growth and crop yield, little information is available on sweetpotato, especially when the plants are grown in combination with high temperature and drought stress. To the best of our awareness, the present study is the first to evaluate the interactive effects of high T, DS, and eCO_2_ on the growth and development of three sweetpotato cultivars. Here, we compare the performance of three sweetpotato cultivars for varying climatic conditions by changing the temperature, moisture content, and CO_2_. We hypothesized that sweetpotato growth and development would be modified by the individual (T or DS) and combined T and DS under ambient and eCO_2_. Hence, the objective of this study was to examine the phenotypic responses of sweetpotato cultivars to high T, DS, and eCO_2_ alone and in combination.

## 2 Materials and methods

### 2.1 Experimental conditions

This study was conducted in sunlit plant growth chambers known as Soil-Plant-Atmosphere-Research (SPAR) units located at the Rodney Foil Plant Science Research facility, Mississippi State University, Mississippi State, MS, United States (33°28′ N, 88°47′ W). “Beauregard” and ‘Hatteras’ slips were obtained from the Pontotoc Ridge-Flatwoods Branch Experiment Station, Pontotoc, MS, and “Louisiana 1188” (LA1188) slips were obtained from the Louisiana State University Sweetpotato Research Station, Chase, LA. Sweetpotato slips (vine tip cuttings) were planted into polyvinyl chloride pots (20 cm diameter and 35 cm high) and were filled with a 1:3 (v/v) mix of topsoil and sand on 15 July 2016. Plants were irrigated three times daily with full-strength Hoagland’s solution (Hewitt, 1952) delivered at 08:00, 12:00, and 16:00 h to maintain good nutrient requirements and soil-water conditions for plant growth through an automated and computer-controlled drip irrigation system. Air temperature, CO_2_, and soil moisture content in each chamber were monitored and adjusted every 10 s. The daytime temperature was initiated at sunrise and returned to the nighttime temperature 1 h after sunset. The relative humidity of each chamber was monitored with a sensor (HMV 70Y, Vaisala Inc., San Jose, CA) installed in the return air duct. The vapor pressure deficit (VPD) was calculated based on the percentage-of-evapotranspiration (ET) values recorded the previous day (Murray, 1967) of water provided to each for treatment. The specification and details of SPAR operation and control algorithms have been detailed in Reddy et al. (2001). The chambers consisted of an upper Plexiglas unit 2.5 m tall by 2 m long by 1.5 m wide and a lower steel soil bin 1 m deep by 2 m long by 0.5 m wide. The chambers allowed 97% of the visible solar radiation to pass without spectral variability in absorption while blocking solar UV radiation (100% of UV-B and 88% of UV-A). During the experiment, the ambient solar radiation measured with a pyranometer (Model 4-48, The Eppley Laboratory Inc., Newport, Rhode Island, United States) was 21.26 ± 0.5 MJ m^−2^ d^−1^. Variable density shade cloth was placed around the edges of plants at transplanting. It was adjusted regularly to match plant heights, simulating natural shading by other plants, and eliminating the need for border plants.

### 2.2 Treatments

Eight SPAR chambers were utilized for this experiment, one per treatment. All environmental treatments were imposed at planting except soil moisture treatments, which were set at 36 DAP and continued through the termination of the study at 83 DAP (Table 1). The pots were randomly arranged within each unit to avoid the positional effect. In this study, 144 plants (3 cultivars × 8 treatments × 6 replications) were used to explore temperature (30/22°C day/night, control and 38/30°C day/night, high temperature, T), 2 levels of CO_2_ (410 ppm, CO_2_ and 760 ppm, eCO_2_), 2 levels of soil moisture (100%, control and 50% ET, drought stress-DS), and three (“Beauregard,” “Hatteras,” and “LA1188”) different sweetpotato cultivars (CUL).

**TABLE 1:** The treatments mean day and night temperatures, daytime CO_2_, and day and night soil moisture content for each unit. Temperature (T), carbon dioxide concentration (CO_2_), elevated CO_2_ (eCO_2_), drought (DS), and cultivars (CUL). Values in the mean ± standard deviation of the mean.

Treatment	Temperature (°C)	CO_2_ concentration (ppm)	Soil moisture content (m^3^ m^-3^)
Control	26.54 ± 0.08	416.76 ± 0.96	0.221 ± 0.01
Drought stress (DS)	25.26 ± 0.10	418.81 ± 0.79	0.142 ± 0.02
High temperature (T)	31.25 ± 0.12	417.25 ± 0.70	0.187 ± 0.01
Elevated CO_2_ (eCO_2_)	26.65 ± 0.21	759.39 ± 1.03	0.218 ± 0.005
DS+T	32.40 ± 0.08	441.48 ± 1.26	0.154 ± 0.02
T+eCO_2_	31.70 ± 0.08	734.74 ± 2.34	0.216 ± 0.02
DS+eCO_2_	25.11 ± 0.07	757.96 ± 1.19	0.150 ± 0.03
DS +T+eCO_2_	32.23 ± 0.09	757.92 ± 1.04	0.149 ± 0.02

### 2.3 Data collection

#### 2.3.1 Gas exchange and fluorescence

At 78 DAP, leaf net photosynthesis (Pn), stomatal conductance (gs), transpiration (Tr), electron transport rate (ETR), and chlorophyll fluorescence (Fv′/Fm′) measurements were taken on the youngest, fully expanded primary vine leaf between 10:00 and 13:00 h using a portable photosynthesis system with an integrated fluorescence chamber head (Li-COR 6400 leaf chamber fluorometer). The temperature and internal CO_2_ concentration were set to the respective environments in each SPAR chamber. While measuring photosynthesis, the photosynthetically active radiation (PAR) was set to 1500 μmol m^−2^ s^−1^ (6400-02 LED light source), and the relative humidity inside the cuvette was maintained at approximately 50%. Fluorescence measurements were taken using the in-built leaf chamber fluorometer, which used two red LEDs with a center wavelength of 630 nm and detector radiation of 715 nm in the photosystem-II fluorescence band. A flash of light >7,000 mmol m^−2^ s^−1^ was achieved using 27 red LEDs to measure the maximal fluorescence (Fm′). The rapid dark adaptation to measure minimal fluorescence (Fo′) was performed by turning off the actinic light using the far-red LED with a center wavelength of 740 nm. The software in the instrument provided data on the Pn, gs, Tr, and calculated the quantum efficiency by open photosystem-II reaction centers in the light as the ratio of variable/maximal fluorescence (Fv′/Fm′). Plant water use efficiency (iWUE) was calculated as the ratio between Pn and Tr.

#### 2.3.2 Photosynthetic pigments

Photosynthetic pigments such as chlorophyll a, chlorophyll b, and carotenoid content were measured at 78 DAP. Within each chamber, three leaves from each of the six plants were collected. Discs with 2.0 cm^2^ size were cut from leaves and placed in vials containing 5 ml of dimethyl sulphoxide for chlorophyll extraction. The absorbance of the supernatant was measured with a Bio-Rad ultraviolet/VIS spectrophotometer (Bio-Rad Laboratories, Hercules, CA) at 470, 648, and 663 nm. Total chlorophyll and carotenoid content were estimated (Lichtenthaler, 1987; Chappelle et al., 1992) and expressed on a leaf area basis (µg cm^−2^).

#### 2.3.3 Relative cell membrane injury

The leaf cell membrane thermostability was evaluated at 78 DAP (Martineau et al., 1979). Leaf samples were collected as described in 2.3.2. Eighteen leaf discs with 1.3 cm^2^ were placed in test tubes with 10 ml of deionized water. The leaf discs were thoroughly rinsed three times with deionized water to remove electrolytes, adhering to the leaf surface and leaching from the leaf disc’s cut surfaces. After the final rinse, all the test tubes with leaf discs were filled with 10 mL of deionized water and capped with aluminum foil to prevent the evaporation of water. One set of test tubes was incubated for 20 min at 55°C in a temperature-controlled water bath, whereas the other set was left at room temperature of 25°C. After incubation, the sets of test tubes were brought to 25°C and the initial measurement of the conductance of the control (CEC1) and the treatment (TEC1) was measured by an electrical conductivity meter (Corning Checkmate II; Corning Inc., Corning, NY) at room temperature. Tubes were then autoclaved at 0.1 MPa for 12 min to kill tissues completely, releasing all the electrolytes. Tubes were then cooled to 25°C, and final conductance was measured (CEC2 and TEC2). Finally, the ratio of the initial to the last electrolyte leakage caused by the elevated temperature was estimated, giving a measure of the extent of damage to cellular membranes using the following equation.

$$Cell\;membrane\;thermostability\;\left(\%\right)=\left[1\;–\left(TEC1/TEC2\right)\right]/\;\left[1\;–\left(CEC1/CEC2\right)\right]x100$$

Finally, the inverse value of cell membrane thermostability was used to determine the relative cell membrane injury (RI) across stress treatments.

$$Relative\;cell\;membrane\;injury\;\left(RI,\;\%\right)=\left[1/cell\;membrane\;thermostability\right]$$

#### 2.3.4 Growth and development

Replicated plants from each cultivar per chamber were evaluated for main vine length, measured from the soil surface to the most recently unfolded leaf and node number. Above-ground plant parts were separated (stems and leaves) to record weights. The leaf area was measured using the LI-COR leaf area meter (LI-3100: LI-COR, Inc., Lincoln, NE). According to Meyers et al., 2017, the below-ground plant portions were removed from pots and separated into storage, pencil, and fibrous roots. The number and fresh weight of each type of root were recorded. All plant parts were dried in a forced-air oven at 75°C for 72 h to estimate plant-component dry weights.

### 2.4 Data analysis

The measured parameters were subjected to factorial ANOVA using JMP software (Statistical Institute, Inc., Cary, NC). Summary of source of variance, degrees of freedom and sum of squares of all traits are in given Supplementary Table S1. In the present study, stress response index (SRI) values for all measured were calculated for each treatment (DS or T or eCO_2_ or DS + T or T + CO_2_ or T + CO_2_ or DS + CO_2_ or DS + T + CO_2_) as the value of a parameter (Pl) for a given cultivar at the stress divided by the value of the same parameter at the control (Po). The degree of tolerance among cultivars or parameters responses was assessed by comparing the cultivar relative to their control treatment. The genotypes with a high value indicate better performance in response to treatment.

$$Stress\;response\;index\;\left(SRI\right)=Pl/Po$$

SRI values calculated across cultivars were to evaluate the vegetative, physiological, and biochemical responses of sweetpotato to the treatments under study (eCO_2_, T, DS, DS + T, T + _e_CO_2_, DS + eCO_2_, DS + T + eCO_2_). Traits with high or low values of SRI indicate positive or negative responses to treatment, respectively. Finally, SRI values were used to identify a set of individual and interactive stress tolerance traits in sweetpotato. Graphical analysis was performed with SigmaPlot 14.5 (Systat Software Inc., San Jose, CA).

## 3 Results

### 3.1 Gas exchange parameters

**FIGURE 1:**
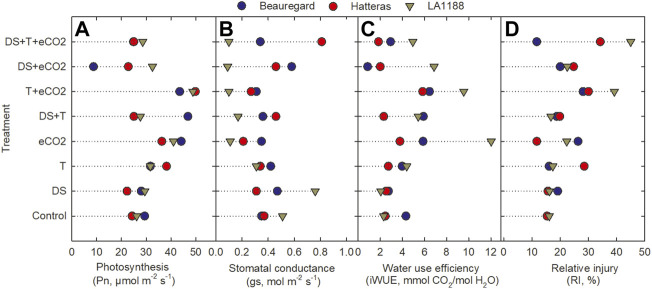
Effects of temperature (T), carbon dioxide concentration ([CO_2_]), drought (DS), and their interaction on **(A)** photosynthesis rate, **(B)** stomatal conductance, **(C)** water use efficiency and **(D)** relative injury of the cell membrane (RI) of three sweetpotato cultivars (Beauregard, Hatteras, and LA 1188). Each data point indicates the mean ± standard error.

The effects of DS, T, eCO_2,_ cultivar (CUL) × eCO_2_, DS × eCO_2_, CUL × DS × eCO_2_, and CUL × T × DS interactions had a significant impact on net photosynthesis (Table 2). Averaged across all cultivars, eCO_2_ alone or combinations with the high T had a 52% (eCO_2_) to 78% (T + eCO_2_) increase in Pn (Figure 1A). Photosynthesis of LA1188 increased across all treatments compared to control (Figure 1A, Supplementary Table S2). Cultivar Beauregard recorded the highest reduction in Pn under DS + eCO_2_ (70%), followed by DS + eCO_2_+T treatments (15%) compared to control (Table 3). Averaged across cultivar, the eCO_2_ alone and in combination with T or DS decreased the gs (46%, 45%, and 9%) and Tr (33%, 27%, and 16%) in comparison with control (Supplementary Table S3). The gs and Tr of Hatteras decreased under DS, while the iWUE was increased (Figures 1B, C). Although some treatment effects were non-significant, iWUE increased across treatments except for DS (Table 2). The individual (eCO_2_, DS, and T) and combined (DS + eCO_2_, DS + T + eCO_2_) treatments significantly affected the iWUE and chlorophyll fluorescence (Fv'/Fm') measurements. Across cultivars, a substantial reduction in electron transport rate and Fv'/Fm' were observed in four out of six treatments (eCO_2_, DS + T, T + eCO_2,_ and DS + eCO_2_). An increase in iWUE was associated with decreased gs and Tr under eCO_2_, DS + T, T + eCO_2_, D + eCO_2,_ and DS + T + eCO_2_ than control in LA 1188 (Figure 1C).

**TABLE 2:** The analysis of variance across the treatments of temperature (T), carbon dioxide concentration ([CO_2_]), drought (DS), and cultivars (CUL), and their interactions on sweetpotato vegetative, physiological, and photosynthetic parameters.

Source of variation	Pn	gs	Tr	iWUE	Fv'/Fm'	ETR	CHL	CARO	RI	VL	NN	LA	LW	SW	RW	SRN	PRN	SRFW	SRW
CUL	NS	*	**	***	NS	NS	*	NS	NS	***	NS	NS	NS	NS	***	**	*	NS	NS
CO_2_	**	*	*	***	*	NS	NS	**	***	NS	NS	NS	NS	***	**	**	*	*	*
DS	***	*	NS	***	***	NS	*	NS	NS	***	**	**	***	***	**	NS	NS	NS	NS
T	***	NS	NS	*	NS	NS	NS	NS	***	*	***	NS	NS	*	NS	**	***	***	***
CUL × eCO_2_	**	**	**	***	***	**	NS	NS	*	NS	NS	NS	NS	NS	NS	NS	NS	NS	NS
CUL × DS	NS	NS	NS	NS	NS	NS	NS	NS	NS	NS	NS	NS	NS	NS	NS	NS	NS	NS	NS
CUL × T	NS	*	*	NS	NS	NS	NS	NS	**	*	NS	*	NS	NS	NS	*	NS	**	**
CO_2_ × DS	***	NS	NS	***	*	NS	**	NS	NS	NS	NS	NS	NS	NS	NS	NS	*	NS	NS
T × eCO_2_	NS	NS	NS	*	**	*	NS	NS	*	NS	NS	**	*	NS	NS	NS	NS	NS	NS
T × DS	NS	NS	NS	NS	NS	NS	NS	NS	NS	NS	NS	NS	NS	NS	NS	NS	*	NS	NS
CUL × DS × eCO_2_	**	NS	NS	NS	NS	NS	NS	NS	**	NS	NS	NS	NS	NS	NS	NS	NS	NS	NS
CUL × T × eCO_2_	NS	*	*	**	NS	NS	NS	NS	*	NS	NS	NS	NS	NS	NS	NS	NS	NS	NS
CUL × T × DS	**	NS	NS	NS	NS	NS	NS	NS	NS	NS	NS	NS	NS	NS	NS	NS	NS	NS	NS
T × DS × eCO_2_	NS	NS	NS	NS	NS	NS	*	NS	NS	NS	NS	NS	NS	NS	NS	NS	NS	NS	NS
CUL × T xDS × eCO_2_	NS	NS	NS	NS	NS	*	NS	NS	NS	NS	NS	NS	NS	NS	NS	NS	NS	NS	NS

Photosynthesis (Pn, μmol m^−2^ s^−1^), stomatal conductance (g_s_, mol m^−2^ s^−1^), transpiration (Tr, mmol H_2_O m^−2^ s^−1^), instantaneous water use efficiency (iWUE, μmol m^−2^ s^−1^/mmol H_2_O m^−2^ s^−1^), quantum efficiency (Fv'/Fm'), electron transport rate (ETR, µmol electrons m^−2^s^−1^), chlorophyll (CHL, μg cm^−2^), carotenoids (CARO, μg cm^−2^), relative cell membrane injury (RI, %), longest vine length (VL, cm), node number (NN, number), leaf area (LA, cm^2^), leaf dry weight (LW, g plant^−1^), stem dry weight (SW, g plant^−1^), root dry weight (RW, g plant^−1^), storage root number (SRN, number), pencil root number (PRN, number), storage root fresh weight (SRFW, g plant^−1^), storage root weight (SRW, g plant^−1^). Elevated carbon dioxide concentration (eCO_2_). †Significance levels are indicated by ***, **, * and NS, representing *p* < 0.001, *p* < 0.01, *p* < 0.05 and *p* > 0.05, respectively.

**TABLE 3:** Percentage changes in the vegetative, physiological, and photosynthetic parameters of three sweetpotato cultivars (drought, DS; high temperature, T; elevated CO_2_, eCO_2_) and their interactions. Data for each cultivar are the mean (*n* = 6). Values indicate treatment-induced decrease (“−”) or increase (“+”) compared to control.

Treatment	Cultivar	Pn	gs	Tr	iWUE	Fv'/Fm'	ETR	CHL	CARO	RI	VL	NN	LA	LW	Sw	RW	SRN	PRN	SRFW	SRW
DS	Beauregard	−4.7	33.0	48.9	−36.5	27.7	72.1	7.8	13.0	24.7	−35.5	−23.1	−46.0	−40.5	−52.5	−64.3	−34.2	−75.5	1.6	1.5
	Hatteras	−8.4	−16.3	−10.3	3.5	−3.2	3.3	17.7	20.1	2.0	−36.4	−20.2	29.2	−37.0	−47.2	27.0	2.6	15.4	66.9	69.4
	LA 1188	12.6	48.6	−4.3	−11.5	4.2	−9.4	3.1	10.3	0.2	−40.0	−29.0	7.4	21.9	−40.5	−48.6	−30.8	0.0	−9.8	−18.7
T	Beauregard	7.9	21.0	23.0	−7.9	5.6	17.0	−14.8	−13.0	4.8	−11.8	16.7	−21.9	−15.5	3.1	−44.0	−69.3	−6.9	−61.2	−54.4
	Hatteras	57.2	−7.3	38.8	11.6	−19.9	3.5	5.9	15.8	85.8	25.9	23.7	119.7	16.6	13.2	8.8	0.0	115.4	28.9	68.5
	LA 1188	21.0	−39.4	−32.8	90.0	−8.3	−10.4	7.7	3.3	7.6	−8.1	3.8	−62.3	−48.1	−16.7	−49.0	−42.3	94.1	−73.3	−62.7
CO_2_	Beauregard	50.3	0.9	16.3	35.8	6.8	4.7	0.7	−15.6	71.6	−19.3	6.5	−29.9	3.2	10.5	−42.1	−16.7	−75.5	9.2	8.5
	Hatteras	49.2	−43.4	−1.9	54.3	−12.7	20.7	12.8	2.0	−23.8	−7.2	−9.1	122.7	42.6	62.0	109.3	−7.9	115.4	−24.2	−20.1
	LA 1188	56.8	−79.3	−77.3	419.7	−38.8	−69.3	26.8	16.7	38.4	−20.5	−1.7	−21.9	18.8	38.8	12.7	11.5	64.7	61.6	54.1
DS + T	Beauregard	59.4	3.0	16.5	36.5	6.3	−5.4	−6.1	−8.3	22.4	−32.9	−0.3	−69.5	−57.6	−36.6	−48.5	−62.7	66.7	−82.6	−77.0
	Hatteras	3.0	26.0	30.1	−5.3	−6.4	31.4	−16.7	−11.8	29.3	−6.8	17.8	−26.9	−55.2	−23.5	−17.5	10.5	130.8	41.3	37.2
	LA 1188	5.7	−67.0	−66.2	134.6	−16.9	−28.1	4.3	−1.1	3.7	−19.7	4.6	−69.3	−56.9	−30.3	−29.5	−61.5	158.8	−92.9	−93.3
T + eCO_2_	Beauregard	48.1	−11.0	7.1	49.1	−6.5	−33.6	−0.2	−14.4	83.1	−15.2	16.0	2.8	17.4	26.5	−4.8	−45.2	17.6	−14.7	5.2
	Hatteras	104.9	−27.3	6.2	138.2	−7.7	36.4	−2.5	−3.7	95.6	22.7	14.6	202.6	22.8	81.4	64.4	5.3	76.9	18.1	11.0
	LA 1188	86.7	−81.5	−65.5	312.4	−28.6	−28.6	5.8	1.4	143.1	24.1	37.7	−44.6	−23.5	41.1	7.6	−11.5	58.8	−48.1	−56.2
DS + eCO_2_	Beauregard	−70.2	64.3	52.0	−80.0	19.0	15.1	−12.8	−21.3	30.3	−16.1	6.8	−50.5	−42.0	−37.8	−35.8	0.9	−80.4	32.2	48.4
	Hatteras	−6.3	26.5	13.1	−18.3	3.8	20.6	−17.5	−20.7	61.3	−30.0	−9.6	−2.6	−48.4	−41.6	17.3	21.1	−53.8	70.0	88.6
	LA 1188	24.4	−83.4	−67.2	196.5	−26.7	−39.5	−9.2	−17.2	39.5	−32.0	−14.8	−60.3	−43.1	−27.4	−14.1	−13.5	−47.1	13.9	8.4
DS + T + eCO_2_	Beauregard	−15.4	−3.0	33.6	−32.1	22.6	35.3	−8.4	−21.9	−23.7	−21.6	16.0	−32.7	−28.3	2.4	−21.2	−51.8	−6.9	−72.6	−62.6
	Hatteras	3.0	121.5	45.1	−24.9	12.8	33.3	−25.2	−17.8	122.8	−5.6	18.4	27.6	−11.2	26.5	38.8	47.4	84.6	100.9	115.3
	LA 1188	9.3	−79.7	−61.5	113.4	−19.6	−22.2	3.6	−4.8	179.0	−18.9	2.6	−58.4	−28.7	4.7	−7.7	−5.8	11.8	−57.3	-53.5

Photosynthesis (Pn, μmol m^−2^ s^−1^), stomatal conductance (g_s_, mol m^−2^ s^−1^), transpiration (Tr, mmol H_2_O m^−2^ s^−1^), instantaneous water use efficiency (iWUE, μmol m^−2^ s^−1^/mmol H_2_O m^−2^ s^−1^), quantum efficiency (Fv'/Fm'), electron transport rate (ETR, µmol electrons m^−2^s^−1^), chlorophyll (CHL, μg cm^−2^), carotenoids (CARO, μg cm^−2^), relative cell membrane injury (RI, %), longest vine length (VL, cm), node number (NN, number), leaf area (LA, cm^2^), leaf dry weight (LW, g plant^−1^), stem dry weight (SW, g plant^−1^), root dry weight (RW, g plant^−1^), storage root number (SRN, number), pencil root number (PRN, number), storage root fresh weight (SRFW, g plant^−1^), storage root weight (SRW, g plant^−1^).

### 3.2 Pigments and relative cell membrane injury

Significant effects of eCO_2_ and T were observed for relative cell membrane injury (Table 2). The interaction of CUL × eCO_2_, CUL × T, T × eCO_2_, CUL × DS × eCO_2_, and DS × T × eCO_2_ significantly affected membrane injury (Table 2). Averaged across cultivars, relative injury increased to a greater extent under T + eCO_2_ (2-fold) and DS + T + eCO_2_ (94%) than individual stress treatments. “Hatteras” exhibited 24% higher membrane stability (minor injury) under eCO_2_ than the control (Figure 1D). When subjected to DS + T + CO_2_, ‘Hatteras’ and ‘LA1188’ recorded the maximum membrane injury compared to other treatments (Figure 1D; Table 3). Total chlorophyll content demonstrated a significant response to CUL, DS, and the interactions of eCO_2_ × DS and T × DS × eCO_2_ (Table 2). The CHL increased (9.5%) significantly under DS and decreased under eCO_2_ × DS (13%) and T × DS × eCO_2_ (10%) across cultivars. In response to treatments, cultivars increased the CHL, ranging from 1% (in Beauregard under eCO_2_) to 27% (in LA 1188 under eCO_2_), while leaf carotenoid content of all the cultivars decreased under combined stresses, ranging from 1% (in LA 1188 under DS + T) to 22% in Beauregard under combined treatment, DS + T + eCO_2_ (Supplementary Tables S2, S3).

### 3.3 Vine length, node number

**FIGURE 2:**
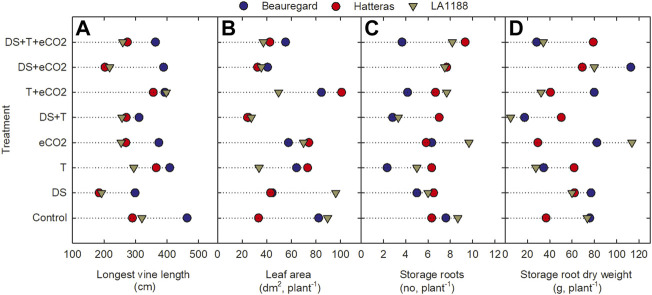
Effects of temperature (T), carbon dioxide concentration ([CO_2_]), drought (DS), and their interaction on **(A)** vine length **(B)** leaf area, **(C)** storage roots, and **(D)** storage root dry weight for three sweetpotato cultivars (Beauregard, Hatteras, and LA1188). Each data point indicates the mean ± standard error.

Significant CUL, DS, T, and CUL × T interactions were observed for vine length, whereas significant treatments (DS and T) impacts were observed for node number (Table 2). DS alone and in combination with eCO_2_ (DS + eCO_2_) resulted in 37% and 25% reductions in the mean value of vine length than control (Figure 2A; Table 3). The combination of T + eCO_2_ resulted in a 23.5% (24% in LA1188 and 23% in Hatteras) increase in main vine length compared to the control (Figure 2A; Supplementary Table S3). Interestingly, high T alone and DS, eCO_2,_ and DS + eCO_2_ positively affected node number among the cultivars (Table 3).

### 3.4 Plant biomass components

Leaf area showed significant responses to DS, CUL × T, and T × eCO_2_ (Table 2). Averaged across cultivars, eCO_2_ in combination with DS decreased the leaf area by 47%, and T increased leaf area by 14% compared to the control (Figure 2B, Supplementary Table S2). The combination of DS + T caused the most significant reduction in leaf area (62%) in all the cultivars. In comparison, ‘Hatters’ had shown a minimum decrease (30%) in leaf area compared to the control (Figure 2B; Table 3). The leaf area of ‘Hatteras’ and “LA1188” was increased significantly under DS compared to the control. ‘Beauregard’ decreased leaf area by 46% under DS than control (Table 3). The shoot and roots’ dry weights were affected by eCO_2_ and DS (Table 3). Averaged across cultivars, eCO_2_ increased leaf weight by 20% (3% in Beauregard; 43% in Hatteras) and shoot dry weight by 34% (11% in Beauregard; 62% in Hatteras) (Table 3). Shoot dry weight declined more with the DS (41% in LA1188 to 53% in Beauregard) than combined the DS + T (23% in Hatters to 37% in Beauregard). Averaged over the cultivars, plants grown under DS + T showed 62%, 57%, and 31% less leaf area, leaf weight, and shoot weight than the control, respectively (Table 3). However, plants grown under eCO_2_ alone or combined with high T showed an overall increase (34% or 47%) in shoot biomass in all the cultivars.

### 3.5 Storage roots and pencil roots

Storage and pencil root number were significantly varied by CUL, T, and eCO_2_ treatments. However, CUL × T and CUL × DS interactions were observed only for storage and pencil root numbers, respectively (Table 2). Sweetpotato cultivars grown under high T conditions exhibited a 40% reduction in their storage root number (Figure 2C) and a 59% increase in pencil roots (Supplementary Table S3). Storage root dry weight ranged from 5 (under T + DS) to 114 g plant^−1^ (eCO_2_). Plants grown under DS + T had the least storage root dry weight (∼24.5 g), while those produced under eCO_2_ (75 g) and eCO_2_ + DS (87 g) had the most (Figure 2D). The reduction of storage root dry weight under T, DS + T, T + eCO_2_, and DS + T + eCO_2_ treatments were 31%, 60%, 20%, and 21%, respectively, compared to the control (Table 3). Cultivar ‘LA1188’ with the highest number of pencil roots, accumulated the lowest storage root weight (5 g) under DS + T, 93% less than the control and the other cultivars. In contrast, cultivar “Hatteras” with high storage root production had a 37% higher storage root weight under DS + T than the control. Individual (eCO_2_ or T) or combined (DS + T, T+ eCO_2_, DS + T + eCO_2_) treatments resulted in more pencil roots in all three cultivars (Supplementary Table S2). Meanwhile, the formation of pencil roots in all three cultivars under DS + eCO_2_ was decreased by 62% compared to the control and increased the storage root weight by 42% (Table 3).

### 3.6 Stress response index

**FIGURE 3:**
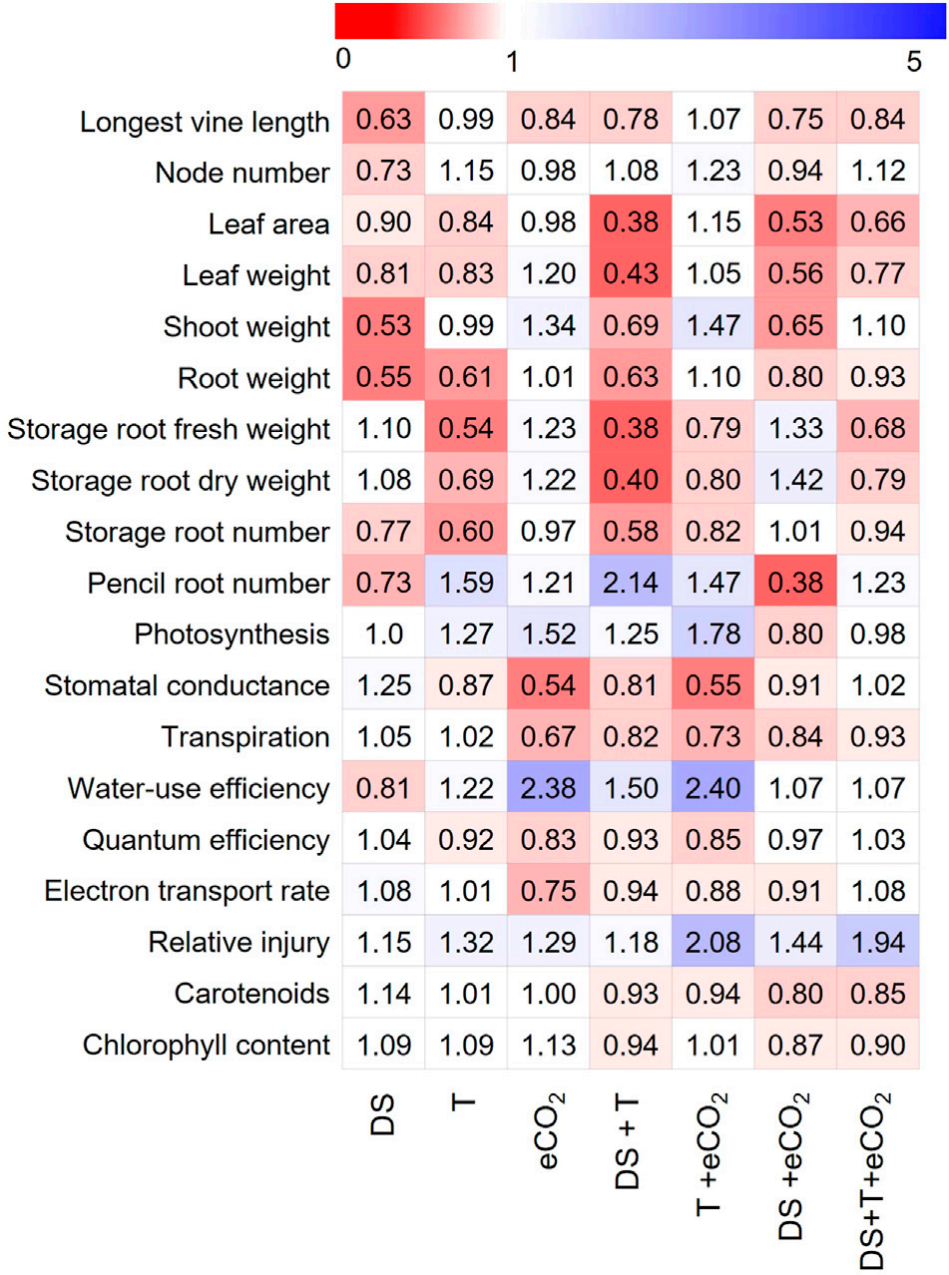
Heat map showing individual and combined treatments induced responses of vegetative, physiological, and photosynthetic parameters relative to control treatment. Each column represents a phenotypic response (average of three cultivars) relative to control (Supplementary Table S3), and each row represents the overall parameter response to treatment. The color codes depict the parameter range for each treatment, with “red” indicating a lower value (<1, decreased compared to control), whereas “blue” indicates a higher value (>1, increased compared to control) for a given parameter. Parameter acronyms and units are given in Table 2.

To understand how vegetative, physiological, and photosynthetic parameters of sweetpotato respond to treatments, the stress response index (SRI) of all measured parameters were calculated across seven treatments (Supplementary Table S3; Figure 3). Among the studied traits, DS had higher adverse effects (see heat map Figure 3, value < 1) on the shoot and root parameters, while eCO_2_ had a favorable impact on root-related parameters. In contrast, DS + T negatively affected gas exchange (except Pn) and pigment parameters. Storage roots were the most severely affected parameters due to high T and combined DS + T. Interestingly, eCO_2_ alone or in combination with DS treatments increased the sink strength or storage root potential up to 42% compared to control (Figure 3). Based on the SRI, cultivars (Beauregard and LA1188) were sensitive to temperature and drought. The impact was much higher when they occurred together (Supplementary Table S3). On average sweetpotato cultivars had a positive response under eCO_2_ conditions, and it weakened the damaging effects of high T and DS in cultivar Hatteras (Table 3).

## 4 Discussion

Sweetpotato is predominately grown in rain-fed conditions or with moderate irrigation at high temperatures (Felix et al., 2015) in the southeastern states of the U.S. and California. Due to this, sweetpotato faces drought or high T during the growing season. On the other hand, a combination of these stresses is reported to occur more often during the growing season. Among all the stages, the storage root formation is most sensitive to drought and high T. Previously studies showed the impact of environmental factors on growth and development of sweetpotato (Pardales and Esquisel, 1996; Gajanayake et al., 2014; Wijewardana et al., 2018). Other studies also reported phenotypic responses of sweetpotato to stressors under ambient CO_2_, such as low temperature (Gajanayake et al., 2014), heat (Heider et al., 2021), and drought (Laurie et al., 2014). However, no studies have reported the multiple stress interactions to date. This is the first study that explored the interactive effect of three major climate change components, such as high T, DS, and eCO_2_, on the growth and development of sweetpotato.

Sweetpotato cultivars used in the study were commonly grown in the southern U.S. and varied in their response to abiotic stresses during storage root formation (Table 3). Sweetpotato cultivars exposed to drought stress during the storage root formation stage (36 DAP) for 47 days caused a more significant reduction in shoot biomass and storage root yield (Figure 3). In agreement with earlier studies (van Heerden and Laurie, 2008; Ravi et al., 2014; Meyers et al., 2017), drought stress decreased the vine length, node numbers, and leaf area (Figures 2, 3). Along the same line, DS and combination with high T reduced the leaf area by 16%–62% compared to control (Figure 3). Based on the stress response index, Beauregard and LA 1188 could not be tolerant of the DS (Supplementary Table S3) or showed a high reduction in phenotypic value (Table 3). At the same time, Hatteras could tolerate drought and high T stress during storage root formation (Table 3). However, none of the cultivars could take combined drought and high-temperature stress, resulting in higher growth limitations and storage root formation (Table 3).

As expected, eCO_2_ positively influenced the leaf area and photosynthesis rate under warmer temperatures (Figure 1). Despite reduced gs, Pn was increased under eCO_2_. Further, our results showed that eCO_2_ could mitigate the negative impact of drought and high T stress, particularly by increasing Pn and decreasing gs (Figure 3). The present study hypothesized that eCO_2_ alone or combined with high T would enhance leaf growth due to the higher photosynthetic rate and iWUE (Figure 3). A potato (*Solanum tuberosum*) survey reported an increase in shoot biomass and WUE under eCO_2_, with the most significant increase occurring under irrigated conditions (Fleisher et al., 2008). Similar to our findings, a nearly 2-fold increase in the WUE under eCO_2_ were observed in alfalfa (De Luis et al., 1999), carrot (Kyei-Boahen et al., 2003), corn (Kim et al., 2006; Wijewardana et al., 2016), cotton (Reddy et al., 1997; Brand et al., 2016), and soybeans (Koti et al., 2005), including potato crops (Lee et., 2020). At the same time, the eCO_2_ alone or in combination with high T increased the biomass and Pn (Figure 3). A positive impact of CO_2_ on storage root weight was observed under DS (Figure 3); thus, dissecting the biochemical or molecular pathways could help develop cultivar tolerant to combined stresses. A similar increase in dry matter production and growth under eCO_2_ has been observed in corn (Wijewardana et al., 2016), soybeans (Koti et al., 2005), and cotton (Reddy and Zhao, 2005; Brand et al., 2016) under sunlit plant growth chamber conditions. An increase in biomass may be due to phenotype modifications; however, an apparent molecular regulation is unknown. The eCO_2_ encouraged higher production of carbohydrates than in ambient CO_2_ possibly be by reducing photorespiration (Nie et al., 1995; Zhao et al., 2019). Also, air and soil temperatures regulate the tradeoff between shoot and storage root growth in sweetpotato (Gajanayake et al., 2013; Heider et al., 2021; Karan and Şanli, 2021; Zhang et al., 2021). Higher soil or air temperatures decrease the production of productive storage roots (Heider et al., 2021). Our findings also suggest that the eCO_2_ may negate the negative effect of high T on the growth and yield of sweetpotato (van Heerden and Laurie, 2008; Heider et al., 2021).

Pigments are the central components of the photosystem complex that are directly linked to photosynthesis and plant productivity. On average, across three cultivars, chlorophyll content increased by 9% and 12% under DS and eCO_2_ compared to control, respectively (Table 3; Figure 3). While it was decreased significantly under interactive DS + eCO_2_ and DS + T + eCO_2_ (Figure 3). The transient increase in the chlorophyll content under DS could be due to reduced leaf area or stress-induced inhibition of cell expansion. About a 14% increase in carotenoids in response to DS confirms high carotenoids' role in protecting the photosystem from oxidative damages (Table 3). Cultivar “Hatteras” with 20% high carotenoid under drought had less cell member injury than the other two cultivars (Table 3). Observed plant responses provide clues for choosing a combination trait for cultivar development, which would perform better in current and projected future climatic conditions.

A distinctive variability was observed among all the treatments or combined with cultivars for storage root growth, development, and pencil root production (Figure 3). The high T-induced increase in photosynthesis and iWUE is associated with lower storage root yield under eCO_2_ (Figure 3). We also observed determinantal effects of high T or combination with eCO_2_ and DS on storage root number and weight under optimum irrigation conditions. In potatoes, higher T delayed tuber initiation and the onset of tuber bulking, and a smaller fraction of dry matter was partitioned to the tubers (van Heerden and Laurie, 2008). The plants grown under warmer conditions produced more pencil roots than DS treatment (Figure 3). Nevertheless, DS, in combination with eCO_2,_ had a beneficial effect on storage root weight under optimum T. Even though the direction of the stress effects was the same for all three cultivars, the magnitude of trait responses varied among cultivars (Figure 3). Our results suggest that the interactive stress effects or predicted climate change would modify the genetic potential of currently growing cultivars. It should be noted that controlled environment experiments may not represent the field production conditions. However, the phenotypic responses of cultivars will inform the physiological, growth, and development processes that are tolerant or sensitive to unfavorable environmental conditions.

## 5 Conclusion

The present study provides evidence on individual and interactive stress effects on sweetpotato plant growth and development. Reduced storage root accumulation and increased penciled roots are seen under high T, T + DS, and DS + T + eCO_2_. Taken together, sweetpotato cultivars are susceptible to DS and high T stresses. Based on the stress response index, Hatteras showed relatively higher tolerance to individual and interactive stress than the other two cultivars. The phenotyping approach and traits responses to individual and interactive stresses in this study could form a basis for further phenotyping of diverse genetic resources and mapping genetic loci to develop climate-resilient sweetpotato. Also, changing climate demands climate-ready varieties with a combination of desirable traits for warming, drought, and eCO_2_ for higher yields. Our findings could be valuable in developing decision-making tools for managing sweetpotato production practices under current and future climatic conditions and developing crop models.

## Data Availability

The original contributions presented in the study are included in the article/Supplementary Material, further inquiries can be directed to the corresponding author.
