# Supplementary material for: Sweetpotato cultivars responses to interactive effects of warming, drought, and elevated carbon dioxide
Source: Front Genet. 2023 Jan 4;13:1080125. doi: 10.3389/fgene.2022.1080125 (PMC9845268; doi:10.3389/fgene.2022.1080125)
Supplement: Supplementary file 1 [file Table1.docx]

Table S1. Sum of squares from analysis for all traits.

| **Source of variation** | **DF** | **Sum of Squares** | | | | | | | | | | | | | | | | | | |
| --- | --- | --- | --- | --- | --- | --- | --- | --- | --- | --- | --- | --- | --- | --- | --- | --- | --- | --- | --- | --- |
|  |  | **Pn** | **gs** | **Tr** | **iWUE** | **Fv'/Fm'** | **ETR** | **CARO** | **CHL** | **RI** | **VL** | **NN** | **LA** | **LW** | **SW** | **RW** | **SRFW** | **SRW** | **SRN** | **PRN** |
| CUL | 2 | 92.2 | 0.28 | 182.9 | 109.8 | 0.00 | 7033.8 | 2.2 | 176.3 | 303.4 | 318572.7 | 210.9 | 3332324.0 | 220.6 | 2012.7 | 209.2 | 64886.4 | 2867.7 | 131.8 | 42.5 |
| CO_2_ | 1 | 257.7 | 0.16 | 68.8 | 59.1 | 0.02 | 6850.6 | 10.7 | 19.3 | 1269.4 | 2240.4 | 616.7 | 4774821.0 | 427.2 | 19827.0 | 86.0 | 358262.1 | 8894.4 | 75.7 | 24.5 |
| DS | 1 | 1897.1 | 0.20 | 6.3 | 67.7 | 0.05 | 6296.0 | 2.3 | 155.9 | 0.5 | 233289.0 | 1534.0 | 237833727.0 | 3927.9 | 37309.5 | 52.4 | 896.7 | 38.5 | 0.9 | 10.8 |
| T | 1 | 734.6 | 0.04 | 0.5 | 9.0 | 0.01 | 271.4 | 2.1 | 92.9 | 803.2 | 61009.0 | 5650.0 | 16386899.0 | 416.0 | 5935.2 | 0.6 | 1806910.0 | 32523.7 | 83.4 | 170.3 |
| CULx eCO_2_ | 2 | 526.1 | 0.58 | 171.4 | 83.3 | 0.09 | 23355.0 | 2.7 | 59.7 | 479.1 | 2054.2 | 478.0 | 64682845.0 | 160.3 | 592.2 | 9.0 | 141002.1 | 3634.1 | 20.0 | 6.9 |
| CULx DS | 2 | 108.9 | 0.12 | 22.3 | 1.7 | 0.01 | 4651.0 | 1.8 | 62.0 | 138.6 | 2308.5 | 380.4 | 36989313.0 | 807.9 | 461.7 | 5.0 | 439976.8 | 9916.7 | 48.6 | 1.4 |
| CUL x T | 2 | 184.5 | 0.34 | 106.6 | 4.1 | 0.00 | 7616.2 | 0.6 | 60.9 | 610.9 | 54608.2 | 431.0 | 162387416.0 | 989.2 | 1195.3 | 7.0 | 1325719.6 | 27487.6 | 106.1 | 3.6 |
| CO_2_ x DS | 1 | 1766.0 | 0.08 | 33.9 | 77.1 | 0.01 | 2279.7 | 5.4 | 247.8 | 1.1 | 21121.8 | 182.3 | 14915708.0 | 207.8 | 950.1 | 0.2 | 208.9 | 907.6 | 21.5 | 32.7 |
| T x eCO_2_ | 1 | 5.0 | 0.09 | 17.0 | 8.4 | 0.03 | 7707.1 | 1.5 | 8.8 | 211.3 | 7980.4 | 8.0 | 100044291.0 | 649.5 | 1954.8 | 20.8 | 4445.9 | 3.3 | 17.1 | 13.8 |
| T x DS | 1 | 8.6 | 0.01 | 6.3 | 2.1 | 0.00 | 988.9 | 0.6 | 0.0 | 72.6 | 11.1 | 84.0 | 15889375.0 | 35.8 | 2740.3 | 20.5 | 70102.5 | 2796.1 | 9.8 | 35.4 |
| CUL x DS x eCO_2_ | 2 | 451.0 | 0.14 | 9.7 | 5.0 | 0.00 | 6852.2 | 0.2 | 2.3 | 581.5 | 27001.7 | 482.8 | 36733744.0 | 182.2 | 1553.6 | 24.4 | 55134.4 | 1938.2 | 0.4 | 11.1 |
| CUL x T x eCO_2_ | 2 | 7.3 | 0.21 | 95.8 | 29.3 | 0.01 | 8120.4 | 0.9 | 63.8 | 377.3 | 8912.4 | 203.1 | 32223714.0 | 202.1 | 1.3 | 15.6 | 29768.8 | 608.1 | 5.9 | 17.1 |
| CUL x T x DS | 2 | 729.1 | 0.18 | 13.9 | 11.3 | 0.01 | 2074.7 | 1.8 | 83.6 | 103.1 | 464.4 | 24.0 | 28470151.0 | 25.2 | 32.4 | 10.7 | 151361.8 | 5282.6 | 2.8 | 0.2 |
| T x DS x eCO_2_ | 1 | 2.4 | 0.03 | 9.8 | 2.5 | 0.00 | 1912.5 | 4.9 | 129.5 | 0.8 | 27335.1 | 380.3 | 10889025.0 | 548.5 | 284.7 | 15.5 | 351.9 | 58.5 | 2.2 | 0.9 |
| CUL x T xDS x eCO_2_ | 2 | 5.2 | 0.04 | 0.3 | 0.3 | 0.01 | 13432.8 | 1.7 | 47.0 | 44.2 | 10214.4 | 320.5 | 12384453.0 | 262.4 | 161.2 | 38.0 | 102413.6 | 4762.7 | 18.3 | 34.8 |

Note- Temperature (T), carbon dioxide concentration ([CO_2_]), drought (DS), and cultivars (CUL). DF- degrees of freedom. Photosynthesis (Pn, µmol m^-2^ s^-1^), stomatal conductance (g_s_, mol m^-2^ s^-1^), transpiration (Tr, mmol H_2_O m^-2^ s^-1^), instantaneous water use efficiency (iWUE, mmol CO_2_/mol H_2_O), quantum efficiency (Fv'/Fm'), electron transport rate (ETR, µmol electrons m^-2^ s^-1^), chlorophyll (CHL, µg cm^-2^), carotenoids (CARO, µg cm^-2^), relative cell membrane injury (RI, %), longest vine length (VL, cm), node number (NN, number), leaf area (LA, cm^2^), leaf dry weight (LW, g plant^-1^), stem dry weight (SW, g plant^-1^), root dry weight (RW, g plant^-1^), storage root number (SRN, number), pencil root number (PRN, number), storage root fresh weight (SRFW, g plant^-1^), storage root weight (SRW, g plant^-1^). Please see *table 2* for significance levels.

Table S2. Effects of the individual (drought, DS; high temperature, T; elevated CO_2_, eCO_2_) and their interactions on sweetpotato vegetative, physiological, and photosynthetic parameters.

| **Treatment** | **Cultivar** | **Pn** | **gs** | **Tr** | **iWUE** | **Fv'/Fm'** | **ETR** | **CHL** | **CARO** | **RI** | **VL** | **NN** | **LA** | **LW** | **SW** | **RW** | **SRN** | **PRN** | **SRFW** | **SRW** |
| --- | --- | --- | --- | --- | --- | --- | --- | --- | --- | --- | --- | --- | --- | --- | --- | --- | --- | --- | --- | --- |
| **Control** | Beauregard | 29.4 | 0.35 | 6.85 | 4.32 | 0.43 | 128.0 | 38.7 | 6.9 | 15.4 | 463.7 | 54.0 | 8218.4 | 31.1 | 81.7 | 8.2 | 7.6 | 3.4 | 491.0 | 75.9 |
|  | Hatteras | 24.3 | 0.37 | 10.08 | 2.45 | 0.50 | 136.2 | 34.6 | 6.0 | 15.4 | 290.5 | 57.0 | 3333.7 | 24.1 | 58.9 | 2.5 | 6.3 | 2.2 | 235.4 | 36.7 |
|  | LA 1188 | 26.1 | 0.51 | 15.02 | 2.31 | 0.57 | 227.3 | 34.6 | 5.8 | 16.1 | 320.3 | 57.5 | 8946.8 | 27.8 | 69.6 | 7.1 | 8.7 | 2.8 | 458.3 | 73.7 |
| **DS** | Beauregard | 28.0 | 0.47 | 10.21 | 2.74 | 0.55 | 220.3 | 41.7 | 7.8 | 19.1 | 298.8 | 41.5 | 4441.2 | 18.5 | 38.8 | 2.9 | 5.0 | 0.8 | 498.7 | 77.0 |
|  | Hatteras | 22.3 | 0.31 | 9.04 | 2.54 | 0.49 | 140.7 | 40.7 | 7.2 | 15.7 | 184.8 | 45.5 | 4308.0 | 15.2 | 31.1 | 3.1 | 6.5 | 2.5 | 393.0 | 62.2 |
|  | LA 1188 | 29.4 | 0.76 | 14.37 | 2.05 | 0.59 | 206.0 | 35.7 | 6.4 | 16.2 | 192.3 | 40.8 | 9612.8 | 33.9 | 41.4 | 3.7 | 6.0 | 2.8 | 413.5 | 59.9 |
| **T** | Beauregard | 31.7 | 0.42 | 8.43 | 3.98 | 0.46 | 149.7 | 33.0 | 6.0 | 16.1 | 408.8 | 63.0 | 6421.8 | 26.3 | 84.2 | 4.6 | 2.3 | 3.2 | 190.6 | 34.6 |
|  | Hatteras | 38.2 | 0.34 | 14.00 | 2.73 | 0.40 | 140.9 | 36.6 | 6.9 | 28.5 | 365.7 | 70.5 | 7324.1 | 28.1 | 66.7 | 2.7 | 6.3 | 4.7 | 303.5 | 61.8 |
|  | LA 1188 | 31.6 | 0.31 | 10.10 | 4.39 | 0.52 | 203.6 | 37.3 | 6.0 | 17.4 | 294.5 | 59.7 | 3370.8 | 14.4 | 58.0 | 3.6 | 5.0 | 5.5 | 122.2 | 27.5 |
| **eCO_2_** | Beauregard | 44.1 | 0.35 | 7.97 | 5.87 | 0.46 | 134.0 | 39.0 | 5.8 | 26.3 | 374.0 | 57.5 | 5758.9 | 32.1 | 90.3 | 4.8 | 6.3 | 0.8 | 536.1 | 82.3 |
|  | Hatteras | 36.3 | 0.21 | 9.89 | 3.78 | 0.44 | 164.3 | 39.0 | 6.1 | 11.7 | 269.5 | 51.8 | 7425.7 | 34.4 | 95.4 | 5.2 | 5.8 | 4.7 | 178.4 | 29.3 |
|  | LA 1188 | 41.0 | 0.11 | 3.41 | 12.02 | 0.35 | 69.8 | 43.9 | 6.8 | 22.3 | 254.7 | 56.5 | 6986.7 | 33.1 | 96.6 | 8.0 | 9.7 | 4.7 | 740.6 | 113.6 |
| **DS+T** | Beauregard | 46.8 | 0.36 | 7.99 | 5.90 | 0.46 | 121.1 | 36.3 | 6.4 | 18.8 | 311.3 | 53.8 | 2509.4 | 13.2 | 51.8 | 4.2 | 2.8 | 5.7 | 85.5 | 17.5 |
|  | Hatteras | 25.0 | 0.46 | 13.12 | 2.32 | 0.47 | 178.9 | 28.8 | 5.3 | 19.9 | 270.7 | 67.2 | 2437.5 | 10.8 | 45.1 | 2.0 | 7.0 | 5.0 | 332.8 | 50.4 |
|  | LA 1188 | 27.6 | 0.17 | 5.08 | 5.43 | 0.47 | 163.4 | 36.1 | 5.7 | 16.7 | 257.2 | 60.2 | 2747.0 | 12.0 | 48.5 | 5.0 | 3.3 | 7.3 | 32.7 | 5.0 |
| **T+eCO_2_** | Beauregard | 43.5 | 0.31 | 7.34 | 6.45 | 0.40 | 85.0 | 38.6 | 5.9 | 28.1 | 393.3 | 62.7 | 8448.2 | 36.5 | 103.3 | 7.8 | 4.2 | 4.0 | 418.9 | 79.9 |
|  | Hatteras | 49.8 | 0.27 | 10.71 | 5.83 | 0.46 | 185.8 | 33.7 | 5.7 | 30.1 | 356.5 | 65.3 | 10086.5 | 29.6 | 106.8 | 4.1 | 6.7 | 3.8 | 278.2 | 40.7 |
|  | LA 1188 | 48.8 | 0.10 | 5.18 | 9.54 | 0.40 | 162.2 | 36.6 | 5.9 | 39.2 | 397.5 | 79.2 | 4956.9 | 21.3 | 98.2 | 7.7 | 7.7 | 4.5 | 237.9 | 32.3 |
| **DS+eCO_2_** | Beauregard | 8.7 | 0.58 | 10.42 | 0.86 | 0.51 | 147.3 | 33.7 | 5.5 | 20.0 | 389.2 | 57.7 | 4068.9 | 18.0 | 50.8 | 5.3 | 7.7 | 0.7 | 649.2 | 112.6 |
|  | Hatteras | 22.8 | 0.46 | 11.40 | 2.00 | 0.52 | 164.3 | 28.5 | 4.7 | 24.8 | 203.3 | 51.5 | 3247.4 | 12.4 | 34.4 | 2.9 | 7.7 | 1.0 | 400.3 | 69.2 |
|  | LA 1188 | 32.5 | 0.09 | 4.92 | 6.86 | 0.41 | 137.6 | 31.4 | 4.8 | 22.5 | 217.8 | 49.0 | 3556.2 | 15.8 | 50.5 | 6.1 | 7.5 | 1.5 | 521.8 | 79.9 |
| **DS +T+eCO_2_** | Beauregard | 24.8 | 0.34 | 9.16 | 2.93 | 0.53 | 173.2 | 35.4 | 5.4 | 11.7 | 363.5 | 62.7 | 5531.4 | 22.3 | 83.6 | 6.5 | 3.7 | 3.2 | 134.4 | 28.4 |
|  | Hatteras | 25.0 | 0.81 | 14.63 | 1.84 | 0.57 | 181.5 | 25.9 | 4.9 | 34.2 | 274.3 | 67.5 | 4254.3 | 21.4 | 74.5 | 3.4 | 9.3 | 4.0 | 473.1 | 79.0 |
|  | LA 1188 | 28.6 | 0.10 | 5.78 | 4.94 | 0.45 | 176.9 | 35.9 | 5.5 | 45.0 | 259.7 | 59.0 | 3720.8 | 19.8 | 72.9 | 6.6 | 8.2 | 3.2 | 195.6 | 34.3 |

Photosynthesis (Pn, µmol m^-2^ s^-1^), stomatal conductance (g_s_, mol m^-2^ s^-1^), transpiration (Tr, mmol H_2_O m^-2^ s^-1^), instantaneous water use efficiency (iWUE, mmol CO_2_/mol H_2_O), quantum efficiency (Fv'/Fm'), electron transport rate (ETR, µmol electrons m^-2^ s^-1^), chlorophyll (CHL, µg cm^-2^), carotenoids (CARO, µg cm^-2^), relative cell membrane injury (RI, %), longest vine length (VL, cm), node number (NN, number), leaf area (LA, cm^2^), leaf dry weight (LW, g plant^-1^), stem dry weight (SW, g plant^-1^), root dry weight (RW, g plant^-1^), storage root number (SRN, number), pencil root number (PRN, number), storage root fresh weight (SRFW, g plant^-1^), storage root weight (SRW, g plant^-1^).

Table S3. Stress response index of three sweetpotato cultivar’s vegetative, physiological, and photosynthetic parameters in response to single (drought, DS; high temperature, T; elevated CO_2_, eCO_2_) and their interactions.

| **Treatment** | **Cultivar** | **Pn** | **gs** | **Tr** | **iWUE** | **Fv'/Fm'** | **ETR** | **CHL** | **CARO** | **RI** | **VL** | **NN** | **LA** | **LW** | **Sw** | **RW** | **SRN** | **PRN** | **SRFW** | **SRW** |
| --- | --- | --- | --- | --- | --- | --- | --- | --- | --- | --- | --- | --- | --- | --- | --- | --- | --- | --- | --- | --- |
| **DS** | Beauregard | 0.95 | 1.33 | 1.49 | 0.63 | 1.28 | 1.72 | 1.08 | 1.13 | 1.25 | 0.64 | 0.77 | 0.54 | 0.60 | 0.48 | 0.36 | 0.66 | 0.25 | 1.02 | 1.01 |
|  | Hatteras | 0.92 | 0.84 | 0.90 | 1.03 | 0.97 | 1.03 | 1.18 | 1.20 | 1.02 | 0.64 | 0.80 | 1.29 | 0.63 | 0.53 | 1.27 | 1.03 | 1.15 | 1.67 | 1.69 |
|  | LA 1188 | 1.13 | 1.49 | 0.96 | 0.88 | 1.04 | 0.91 | 1.03 | 1.10 | 1.00 | 0.60 | 0.71 | 1.07 | 1.22 | 0.59 | 0.51 | 0.69 | 1.00 | 0.90 | 0.81 |
| **T** | Beauregard | 1.08 | 1.21 | 1.23 | 0.92 | 1.06 | 1.17 | 0.85 | 0.87 | 1.05 | 0.88 | 1.17 | 0.78 | 0.84 | 1.03 | 0.56 | 0.31 | 0.93 | 0.39 | 0.46 |
|  | Hatteras | 1.57 | 0.93 | 1.39 | 1.12 | 0.80 | 1.03 | 1.06 | 1.16 | 1.86 | 1.26 | 1.24 | 2.20 | 1.17 | 1.13 | 1.09 | 1.00 | 2.15 | 1.29 | 1.69 |
|  | LA 1188 | 1.21 | 0.61 | 0.67 | 1.90 | 0.92 | 0.90 | 1.08 | 1.03 | 1.08 | 0.92 | 1.04 | 0.38 | 0.52 | 0.83 | 0.51 | 0.58 | 1.94 | 0.27 | 0.37 |
| **eCO_2_** | Beauregard | 1.50 | 1.01 | 1.16 | 1.36 | 1.07 | 1.05 | 1.01 | 0.84 | 1.72 | 0.81 | 1.06 | 0.70 | 1.03 | 1.11 | 0.58 | 0.83 | 0.25 | 1.09 | 1.08 |
|  | Hatteras | 1.49 | 0.57 | 0.98 | 1.54 | 0.87 | 1.21 | 1.13 | 1.02 | 0.76 | 0.93 | 0.91 | 2.23 | 1.43 | 1.62 | 2.09 | 0.92 | 2.15 | 0.76 | 0.80 |
|  | LA 1188 | 1.57 | 0.21 | 0.23 | 5.20 | 0.61 | 0.31 | 1.27 | 1.17 | 1.38 | 0.80 | 0.98 | 0.78 | 1.19 | 1.39 | 1.13 | 1.12 | 1.65 | 1.62 | 1.54 |
| **DS+T** | Beauregard | 1.59 | 1.03 | 1.17 | 1.36 | 1.06 | 0.95 | 0.94 | 0.92 | 1.22 | 0.67 | 1.00 | 0.31 | 0.42 | 0.63 | 0.51 | 0.37 | 1.67 | 0.17 | 0.23 |
|  | Hatteras | 1.03 | 1.26 | 1.30 | 0.95 | 0.94 | 1.31 | 0.83 | 0.88 | 1.29 | 0.93 | 1.18 | 0.73 | 0.45 | 0.77 | 0.82 | 1.11 | 2.31 | 1.41 | 1.37 |
|  | LA 1188 | 1.06 | 0.33 | 0.34 | 2.35 | 0.83 | 0.72 | 1.04 | 0.99 | 1.04 | 0.80 | 1.05 | 0.31 | 0.43 | 0.70 | 0.70 | 0.38 | 2.59 | 0.07 | 0.07 |
| **T+eCO_2_** | Beauregard | 1.48 | 0.89 | 1.07 | 1.49 | 0.94 | 0.66 | 1.00 | 0.86 | 1.83 | 0.85 | 1.16 | 1.03 | 1.17 | 1.26 | 0.95 | 0.55 | 1.18 | 0.85 | 1.05 |
|  | Hatteras | 2.05 | 0.73 | 1.06 | 2.38 | 0.92 | 1.36 | 0.98 | 0.96 | 1.96 | 1.23 | 1.15 | 3.03 | 1.23 | 1.81 | 1.64 | 1.05 | 1.77 | 1.18 | 1.11 |
|  | LA 1188 | 1.87 | 0.18 | 0.35 | 4.12 | 0.71 | 0.71 | 1.06 | 1.01 | 2.43 | 1.24 | 1.38 | 0.55 | 0.77 | 1.41 | 1.08 | 0.88 | 1.59 | 0.52 | 0.44 |
| **DS+eCO_2_** | Beauregard | 0.30 | 1.64 | 1.52 | 0.20 | 1.19 | 1.15 | 0.87 | 0.79 | 1.30 | 0.84 | 1.07 | 0.50 | 0.58 | 0.62 | 0.64 | 1.01 | 0.20 | 1.32 | 1.48 |
|  | Hatteras | 0.94 | 1.26 | 1.13 | 0.82 | 1.04 | 1.21 | 0.82 | 0.79 | 1.61 | 0.70 | 0.90 | 0.97 | 0.52 | 0.58 | 1.17 | 1.21 | 0.46 | 1.70 | 1.89 |
|  | LA 1188 | 1.24 | 0.17 | 0.33 | 2.97 | 0.73 | 0.61 | 0.91 | 0.83 | 1.40 | 0.68 | 0.85 | 0.40 | 0.57 | 0.73 | 0.86 | 0.87 | 0.53 | 1.14 | 1.08 |
| **DS +T+eCO_2_** | Beauregard | 0.85 | 0.97 | 1.34 | 0.68 | 1.23 | 1.35 | 0.92 | 0.78 | 0.76 | 0.78 | 1.16 | 0.67 | 0.72 | 1.02 | 0.79 | 0.48 | 0.93 | 0.27 | 0.37 |
|  | Hatteras | 1.03 | 2.21 | 1.45 | 0.75 | 1.13 | 1.33 | 0.75 | 0.82 | 2.23 | 0.94 | 1.18 | 1.28 | 0.89 | 1.26 | 1.39 | 1.47 | 1.85 | 2.01 | 2.15 |
|  | LA 1188 | 1.09 | 0.20 | 0.38 | 2.13 | 0.80 | 0.78 | 1.04 | 0.95 | 2.79 | 0.81 | 1.03 | 0.42 | 0.71 | 1.05 | 0.92 | 0.94 | 1.12 | 0.43 | 0.46 |

Data for each cultivar are the mean of six plants. The stress response index was calculated as the ratio of stress treatment to control. Traits with high or low values of SRI indicate positive or negative responses to treatment, respectively. Photosynthesis (Pn, µmol m^-2^ s^-1^), stomatal conductance (g_s_, mol m^-2^ s^-1^), transpiration (Tr, mmol H_2_O m^-2^ s^-1^), instantaneous water use efficiency (iWUE, mmol CO_2_/mol H_2_O), quantum efficiency (Fv'/Fm'), electron transport rate (ETR, µmol electrons m^-2^ s^-1^), chlorophyll (CHL, µg cm^-2^), carotenoids (CARO, µg cm^-2^), relative cell membrane injury (RI, %), longest vine length (VL, cm), node number (NN, number), leaf area (LA, cm^2^), leaf dry weight (LW, g plant^-1^), stem dry weight (SW, g plant^-1^), root dry weight (RW, g plant^-1^), storage root number (SRN, number), pencil root number (PRN, number), storage root fresh weight (SRFW, g plant^-1^), storage root weight (SRW, g plant^-1^).
